# Very severe tungiasis in Amerindians in the Amazon lowland of Colombia: A case series

**DOI:** 10.1371/journal.pntd.0007068

**Published:** 2019-02-07

**Authors:** Hollman Miller, Jovana Ocampo, Alvaro Ayala, Julian Trujillo, Hermann Feldmeier

**Affiliations:** 1 Vaupés Health Department, Mitú, Colombia; 2 Grupo SEP Línea de investigación–Interculturalidad, Facultad de Medicina, Universidad de los Andes, Bogotá, Colombia; 3 Department of Neglected Tropical Diseases, Ministry of Health and Social Protection, Bogotá, Colombia; 4 Institute of Microbiology and Infection Immunology, Charité Universitätsmedizin Berlin, corporate member of Freie Universität Berlin, Humboldt-Universität zu Berlin, and Berlin Institute of Health, Berlin, Germany; Hitit University, TURKEY

## Abstract

**Background:**

Tungiasis is a parasitic skin disease caused by penetrating female sand fleas. By nature, tungiasis is a self-limiting infection. However, in endemic settings re-infection is the rule and parasite load gradually accumulates over time. Intensity of infection and degree of morbidity are closely related.

**Methodology/principal findings:**

This case series describes the medical history, the clinical pathology, the socio-economic and the environmental characteristics of very severe tungiasis in five patients living in traditional Amerindian communities in the Amazon lowland of Colombia. Patients had between 400 and 1,300 penetrated sand fleas. The feet were predominantly affected, but clusters of embedded sand fleas also occurred at the ankles, the knees, the elbows, the hands, the fingers and around the anus. The patients were partially or totally immobile. Patients 1 and 3 were cachectic, patient 2 presented severe malnutrition. Patient 3 needed a blood transfusion due to severe anemia. All patients showed a characteristic pattern of pre-existing medical conditions and culture-dependent behavior facilitating continuous re-infection. In all cases intradomiciliary transmission was very likely.

**Conclusion/significance:**

Although completely ignored in the literature, very severe tungiasis occurs in settings where patients do not have access to health care and are stricken in a web of pre-existing illness, poverty and neglect. If not treated, very severe tungiasis may end in a fatal disease course.

## Author summary

Tungiasis (also called sand flea disease) is a neglected tropical disease (NTD) caused by the penetration of female sand fleas in the skin, typically at the toes, the sole or the heel. Once embedded in the upper strata of the skin, the parasite hypertrophies, enlarging its body size by a factor of 2000 within ten days. This causes intense inflammation with pain and itching, eventually leading to impaired mobility. During a period of three weeks, eggs are expelled through a tiny opening in the skin. When the last egg has been released into the environment, the parasite shrinks and eventually dies. Hence, by nature tungiasis is a self-limited infection. However, in endemic settings re-infection is the rule and parasite load gradually accumulates over time. Here we report five cases with extremely severe tungiasis in patients with 400 to 1,300 embedded sand fleas. Not only the feet were affected, but clusters of parasites also occurred at the ankles, the knees, the elbows, the hand, the fingers and around the anus. The patients were partially or totally immobile. Two patients were cachectic and one required a blood transfusion. All patients showed a characteristic pattern of pre-existing medical conditions and culture-related behaviour facilitating continuous re-infection.

## Introduction

Tungiasis (sand flea disease), one of the most neglected tropical diseases (NTDs), is caused by female sand fleas (*Tunga penetrans* and more rarely *T*. *trimamillata*) penetrated into the skin. The disease is prevalent in resource-poor communities in South America, the Caribbean, sub-Saharan Africa and Madagascar [1–5]. Children, the elderly and persons with disabilities bear the highest disease burden [6, 7]. Intensity of infection varies widely and correlates to severity of disease [1, 8]. Tetanus is a known sequel in individuals with insufficient immunization [3, 9].

In endemic communities light infections with 5 to 10 embedded sand fleas predominate. [8]. Reports on very severe tungiasis with a hundred or more embedded sand fleas are scanty in the current literature [10–12]. In contrast, in publications from the end of the 19^th^ to the middle of the 20^th^ century, very severe tungiasis was frequently reported [13–26]. Here we report five cases of very severe tungiasis in inhabitants of four Amerindian communities in Vaupés Department, in the Amazon lowland of Colombia. The patients shared a spectrum of pre-existing medical conditions facilitating constant re-infection and presented socio-economic and environmental characteristics which together influenced the development of tungiasis into a life-threatening condition.

## Methods

### Study area

Vaupés Department is situated in the Southeast part of Colombia and covers an area of 54,134 km^2^. Geographically, it belongs to the Amazon basin and is almost completely covered with dense rain forest. The Río Vaupés is an affluent of the Río Negro, a major tributary to the Amazon River. Vaupés Department is inhabited by about 35,000 people of whom 12,000 live in the capital Mitú. At least 220 Amerindian communities are spread all over the department but only a minority can be accessed by boat or small aircraft. Rarely, communities have more than 200 inhabitants. People live from fishing, hunting and collection of edible plants in the forest and subsistence cultivation of cassava (*Manihot esculenta*).

The only hospital of the department is located in Mitú. A couple of primary health care centers are dispersed in the municipalities of Carurú and Taraira. They serve communities which can be reached by forest tracks or boat.

### Patients

During a period of 12 weeks, five patients with very severe tungiasis were observed. Four patients were seen at the emergency unit of Mitú hospital, one in the community she was living in. Patient 1 and 2 were from Wacará (N01°14ʹ45.19ʺ, W07°00ʹ37.20ʺ, patient 3 from Nuevo Pueblo (N00°51ʹ55.01ʺ, W69°33ʹ52.01ʺ), patient 4 from Los Angeles (N0°34ʹ26.31ʺ, W70°07ʹ28.98ʺ), and patient 5 from Puerto Pinilla (N0°55ʹ39.30ʺ, W69°57ʹ33.15ʺ).

Patient 1 was completely immobile and had to be carried in a hammock from her community to the Vaupés River for six hours and from there she was transported by boat to Mitú. Patient 2 had considerable pain while walking and was hobbling slowly on the lateral rim of his feet. Patient 3 and 4 were completely immobile. They were carried in a hammock to a small airstrip by community members and then transported by aircraft to Mitú. Patient 5 was found living in an isolated place at a small tributary of the Vaupés River. As the access was extremely difficult and her condition was relatively good, she was examined and treated at home.

Patients were undressed, the whole body was washed and carefully examined for the presence of embedded sand fleas. The skin was also inspected for signs of bacterial and fungal infection. Severity of tungiasis was determined using a previously established score [27]. Staging was performed according to the Fortaleza classification [28].

Immediately after the examination, the patients were treated topically with NYDA, a formula containing two dimeticone (silicone) oils with low viscosity (Pohl-Boskamp GmbH & Co. KG, Hohenlockstedt, Germany) [29]. Due to its physical mode of action, NYDA is registered as a class II medical device [29]. Treatment with dimeticones is considered as the reference treatment of tungiasis by the Ministry of Health and Social Protection of Colombia.

Affected body areas were carefully wetted with the dimeticone. In areas with hyperkeratotic skin and several layers of sand fleas situated on top of each other, the oil was vigorously rubbed into the skin. The treatment was repeated after 24 hours. In patient 4, an additional application was made 1 week after the initial treatment.

The tetanus-vaccination status was verified and patients were vaccinated against tetanus if necessary. Because of severe anemia, patient 3 received 2 × 250 ml red blood cell concentrate. Patient 1 was treated with oxacillin intravenously (twice one gram per day for nine consecutive days) due to severe bacterial superinfection of lesions at the feet. Patient 2, 4 and 5 were treated with albendazole (400 mg per day for three days) and tinidazole (three tablets of 500 mg per day for two days) to eliminate intestinal helminths. Patient 3 was treated with metronidazole (500 mg every 12 hours for five days), oxacillin (twice one gram per day for nine days), gentamycin intravenously because an infection with gram-negative bacteria was suspected (160 mg every six hours for seven days) and albendazole (400 mg per day for three days). Patients were monitored for up to 15 days and changes in their clinical condition were documented.

Since the patients did not speak Spanish, questions were translated by health assistants speaking the same language as the patients.

### Ethics statement

The study was performed as part of routine health care provided by public health personnel of Vaupés Health Department in Mitú Hospital. The examination of the skin for the diagnosis of tungiasis is part of the routine health care and was carried out with the aim to cure patients from a life-threatening condition. All patients provided oral consent. The objective of the examination as well as the risks and benefits of treatment were explained to each patient and relatives/caregivers present. The examination of minors was made with the authorization and in the presence of at least one of their parents. In accordance with Resolution 008430 of 1993, of the Ministry of Health and Social Protection of Colombia, which regulates research in humans, the study is classified as a low risk study.

## Results

### Living conditions, medical history and clinical findings

Demographic, socio-economic and clinical characteristics are depicted in Table 1. Patient 1 (female, 72 years) lived together with patient 2, her grandson, in a small hut without a solid floor. She was suffering from gonarthrosis for long. Since she could not work anymore, she rested day and night in her hammock. Food was provided by her son once a day, but food shortage was common. Several dogs belonged to the household.

**Table 1:** Demographic, socio-economic and clinical characteristics of the patients.

Characteristic	Patient 1	Patient 2	Patient 3	Patient 4	Patient 5
Age (years)	72	16	69	81	94
Sex	female	male	male	male	female
Ethnicity	Cacua	Cacua	Hupdah	Tuyuca	Siriana
Duration of tungiasis	> 5 years	> 3 years	> 3 years	approx. 2 years	approx. 1 year
Living conditions	extremely poor	extremely poor	extremely poor	extremely poor	extremely poor
Clinical status	cachexia, anemia, dehydration	malnutrition	cachexia, anemia, dehydration	normal	normal
Weight (kg)/height (cm)	35/147	23/142	39/157	33/160	36/149
Predisposing medical condition	gonarthrosis, hyperopia	mental retardation	gonarthrosis, hyperopia	gonarthrosis	impaired mobility due to old age/loss of vision
Number of penetrated sand fleas^a^	approx. 1,000	approx. 250	approx. 1,300	approx. 400	approx. 1,000^b^
clusters of embedded sand fleas	yes	yes	yes	yes	yes
Hyperkeratosis of soles and lateral rim with several layers of sand fleas on top of each other	yes	yes	yes	yes	yes
Ectopic localizations	ankles, knees, elbows, hands, fingers	lower legs, perianal	ankles, elbows, hands, fingers, perianal	elbows, hands knees	elbows, hands
Degree of immobility	completely immobile	difficulty in walking	completely immobile	completely immobile	difficulty in walking
Bacterial superinfection of lesions	yes	yes	yes	yes	yes

^a^ viable and non-viable stages combined

^b^ of which approximately 50 were viable

Patient 2 (male, 16 years) was the grandson of patient 1. He suffered from bilateral deafness and mental retardation since birth and had never left the village he was born in. He was unable to take care for himself or to accompany his father for hunting or gathering food. Instead he waited the whole day inside the hut, crouching on his heels or directly squatting on the ground next to his grandmother. The only piece of clothing he had were torn shorts.

Patient 3 (male, 69 years) lived in a very remote community located near the frontier with Brazil. He belonged to the Iupdah-Maku ethnicity, a group of Amerindians who only recently became sedentary. Iupdah people entirely live from hunting and edible fruits they collect in the rain forest. The patient was suffering from gonarthrosis and therefore was unable to walk. He entirely depended on food provided by relatives. His daughter, who had taken care of him, had moved away to another community a couple of months ago. The patient was left alone in a shelter without walls, where he spent the whole day in the hammock. Two dogs were his only companions.

Patient 4 (male, 81 years) lived in a small community located at the Brazilian border. He belonged to the Tuyuca ethnic group. He suffered from gonarthrosis since long and was cared for by his daughter. However, the daughter had moved away some time ago. The eldest son should have taken care of the patient, but was unable to provide sufficient food even for his own family.

Patient 5 (female, 94 years) lived in an isolated dwelling at a small tributary of the river Vaupés. She belonged to the Siriana ethnic group and lived with her oldest son, who had no wife. Her mobility and vision were restricted. A week before the patient was identified by the medical team, she had been treated by a relative who had applied a plant extract of unknown origin on the feet. This had reduced the number of viable lesion from around 1000 to about 50.

**Fig 1:**
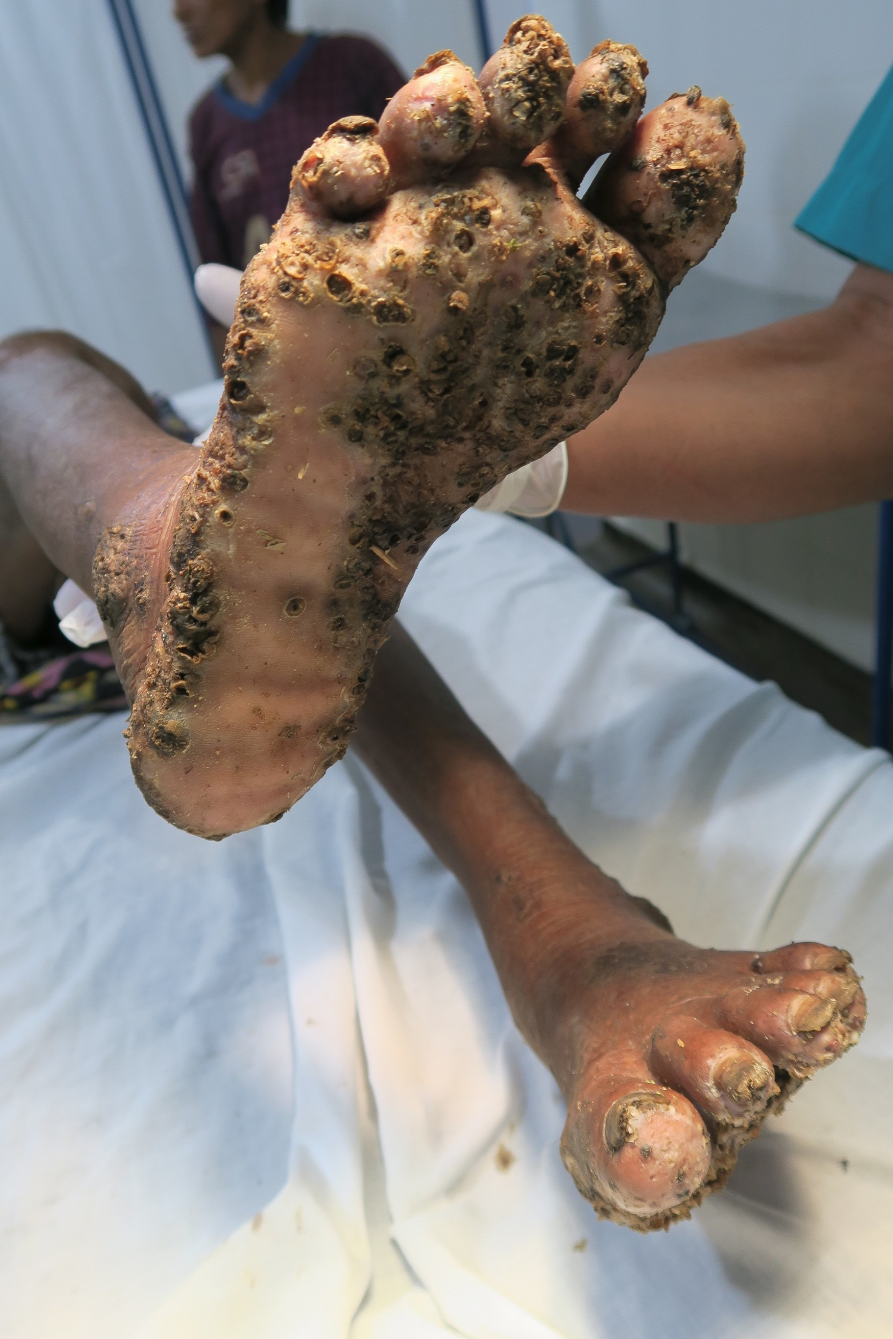
Extremely severe tungiasis at both feet. The soles are covered with several layers of embedded sand fleas on top of each other.

**Fig 2:**
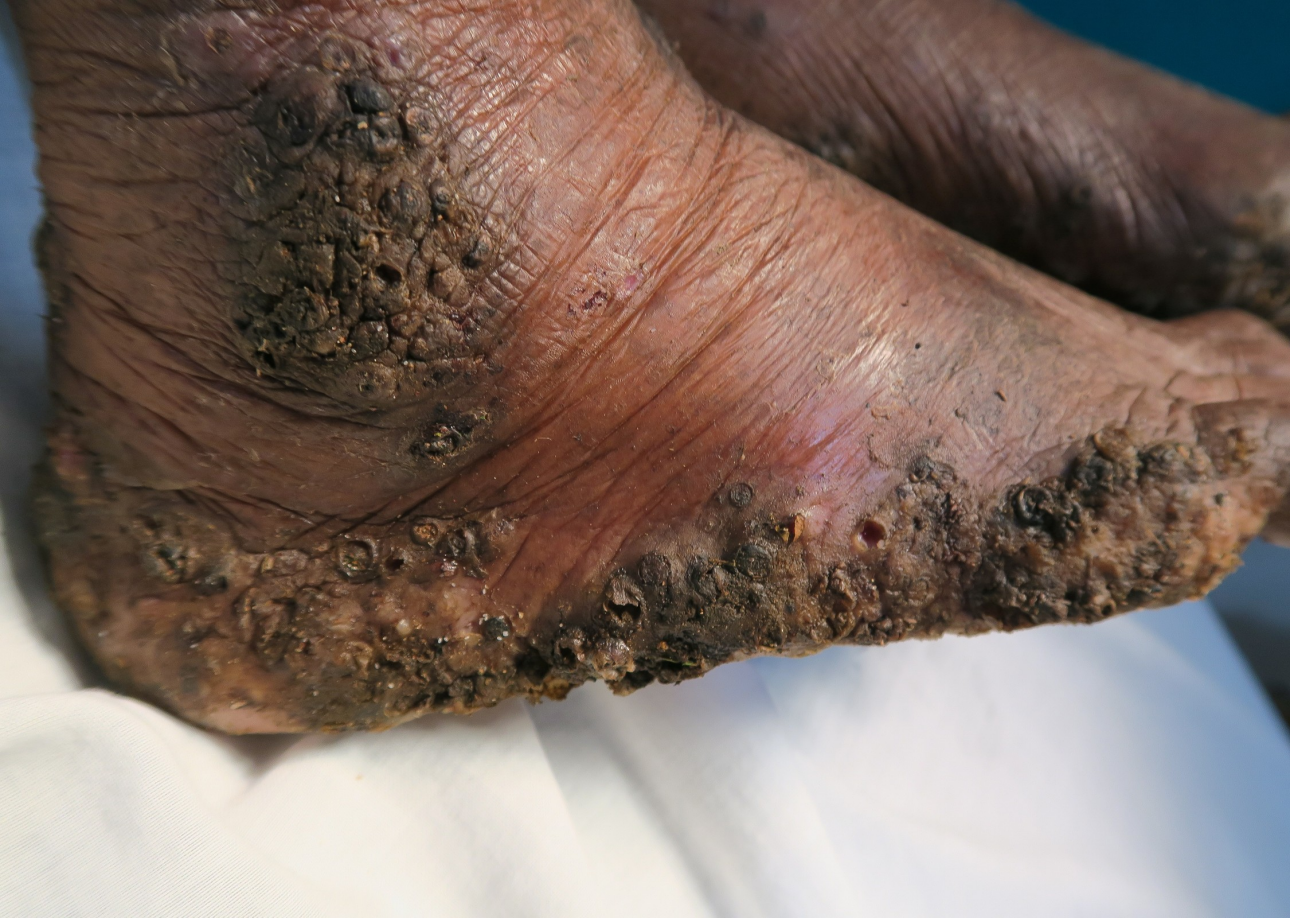
Lateral rim of right foot of the same patient as in Fig 1. Several layers of embedded sand fleas are located on top of each other. A larger cluster of parasites is located at the ankle.

**Fig 3:**
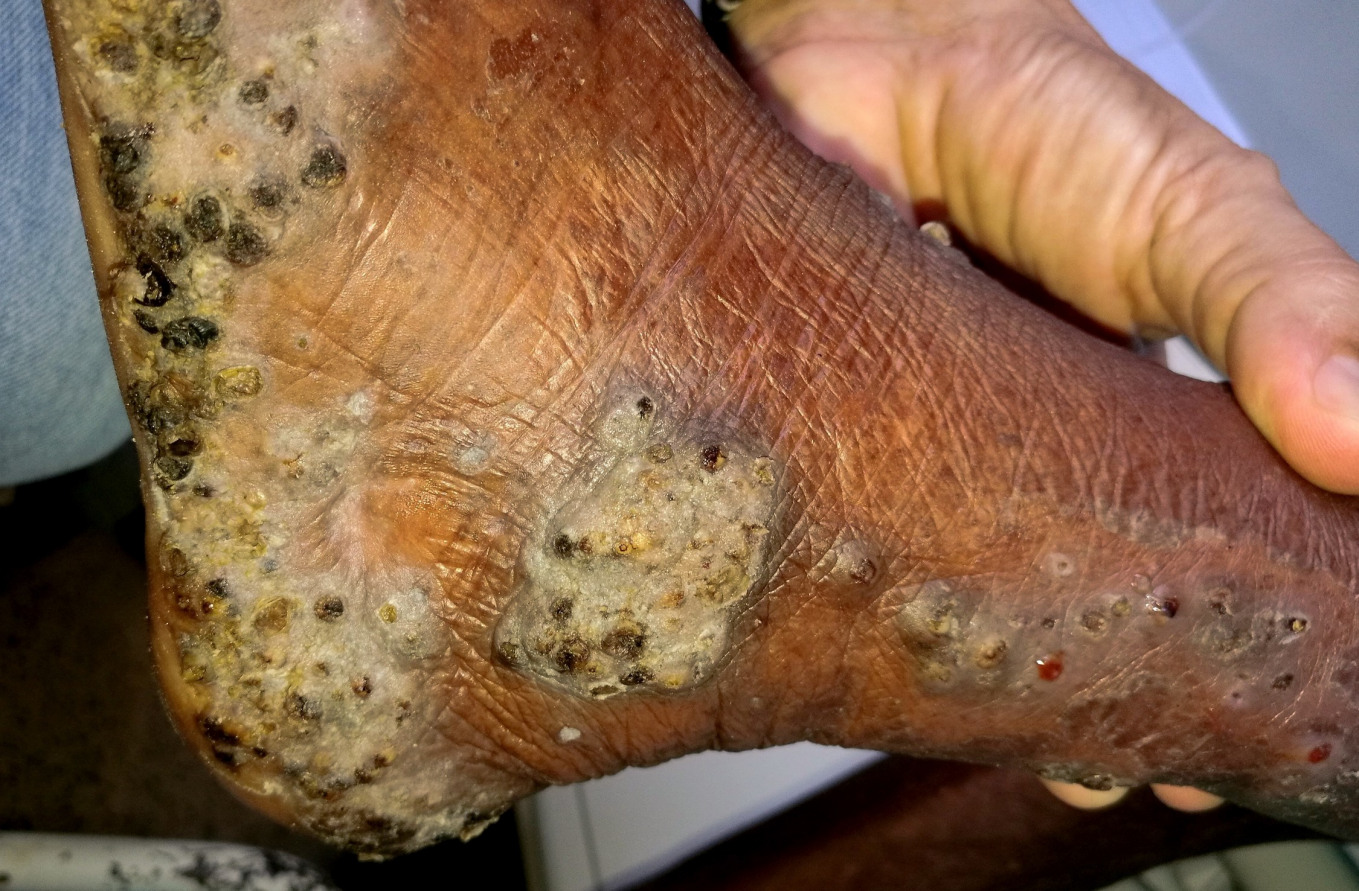
Clusters of embedded sand fleas at the ankle and the lateral side of the lower leg.

**Fig 4:**
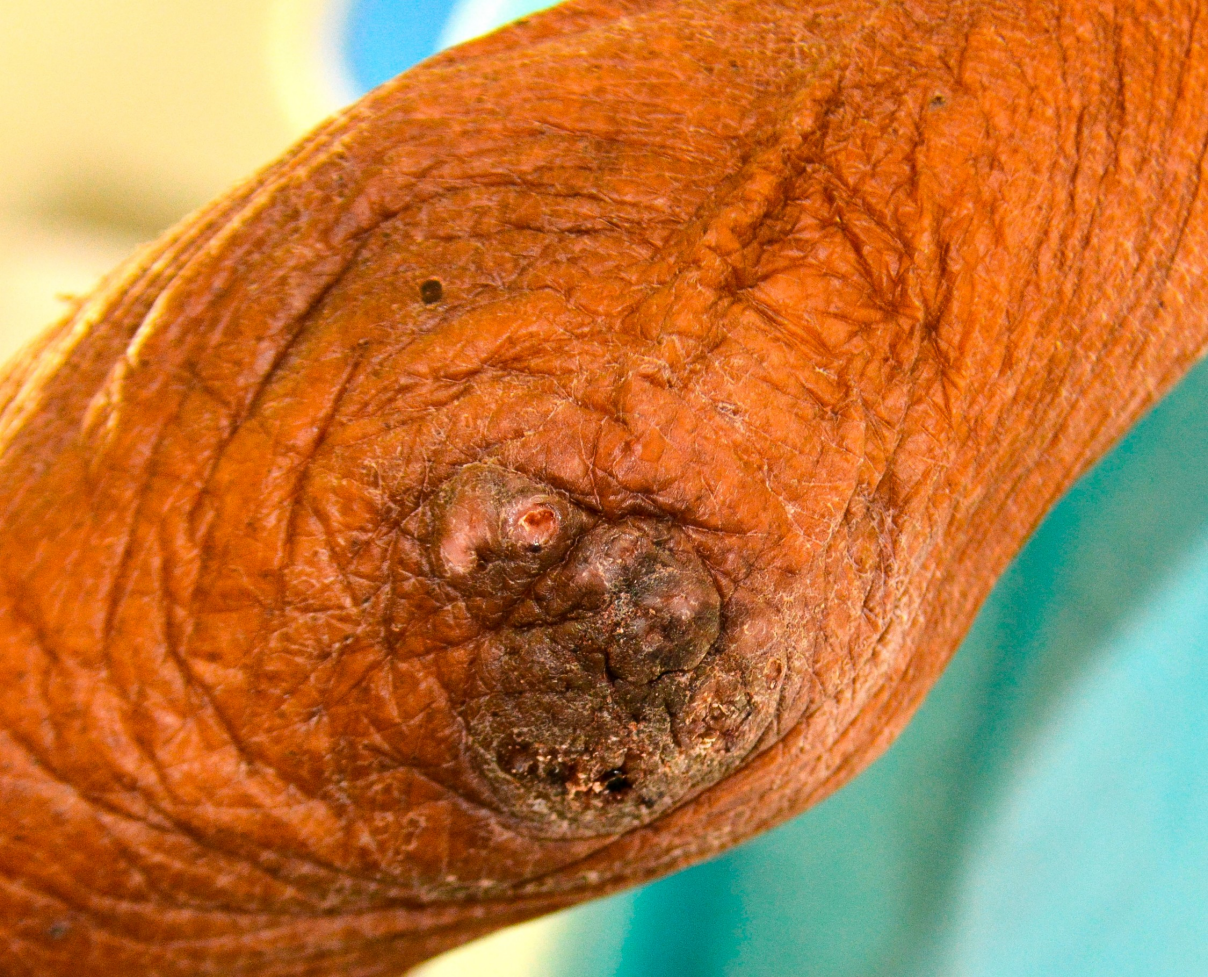
A cluster of embedded sand fleas at the elbow.

**Fig 5:**
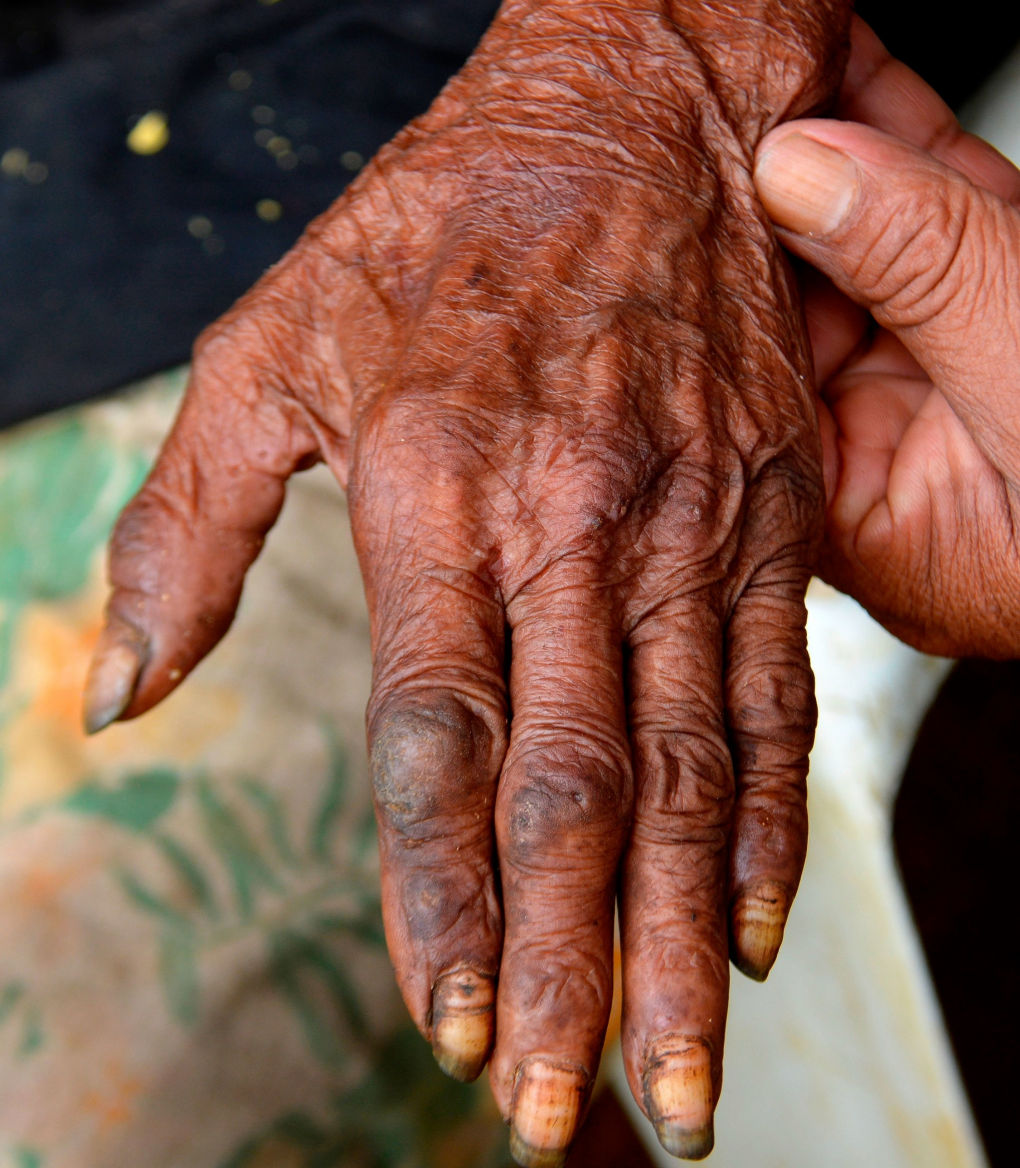
Ectopic localization of embedded sand fleas on the back of the hand.

**Fig 6:**
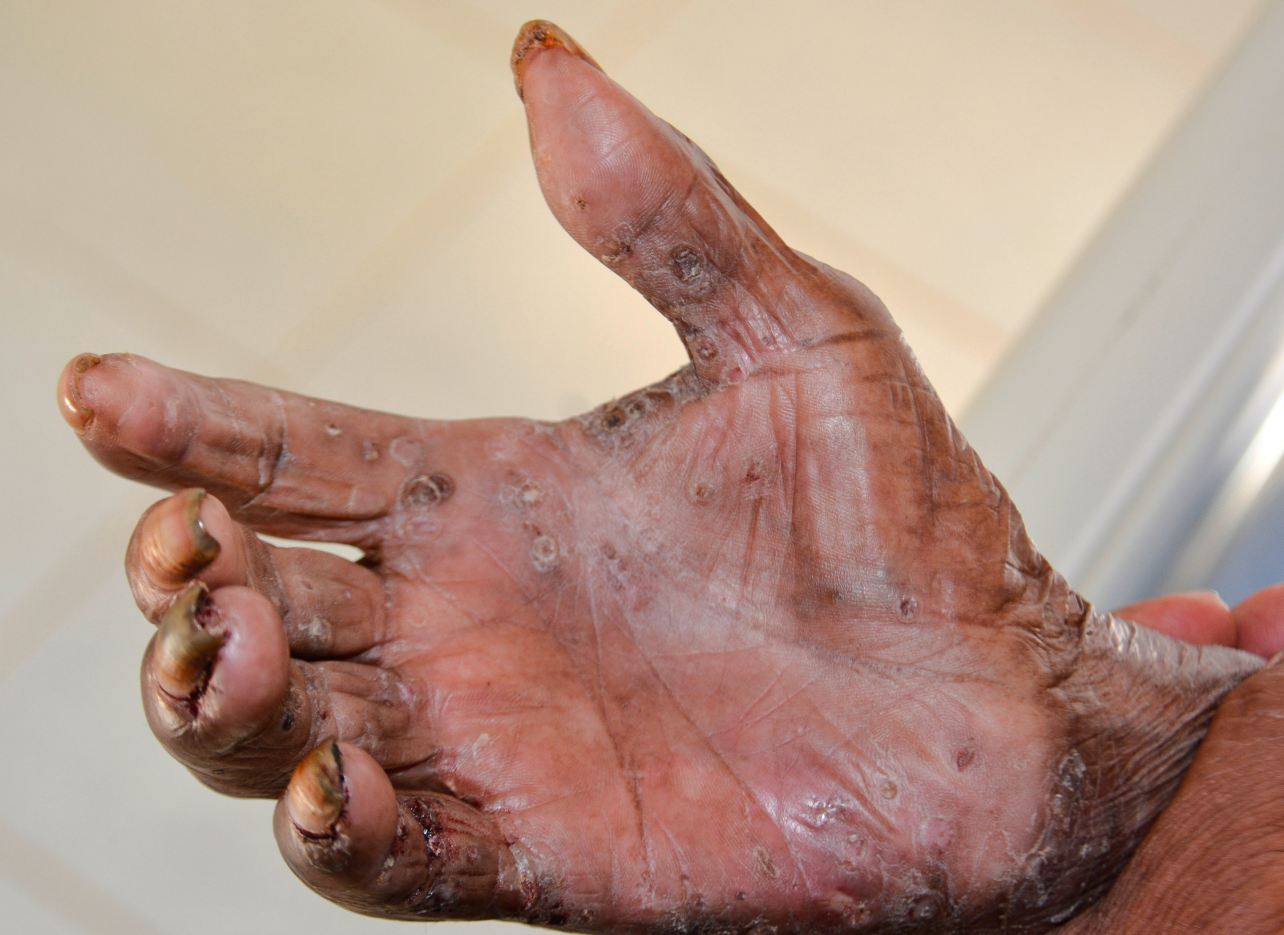
Ectopic localization of embedded sand fleas in the palm of the hand.

Patient 1 and 3 had an extremely severe form of tungiasis with approximately 1,000–1,300 embedded sand fleas in all stages of development. The soles and the lateral rims of both feet were covered with several layers of embedded sand fleas on top of each other and closely-packed (Figs 1 and 2). Clusters of embedded sand fleas existed at the ankles, lower legs, knees, at the elbows and around the anus (Figs 3 and 4). The palm, the back of the hand and the fingers were also affected (Figs 5 and 6). The feet emitted a strong odor of necrotizing flesh. Patient 1 and 3 were anemic, dehydrated and cachectic. Their weight was 35 kg and 39 kg, respectively. Patient 1 was intensely infested with head lice and also had myiasis at the right foot.

**Fig 7:**
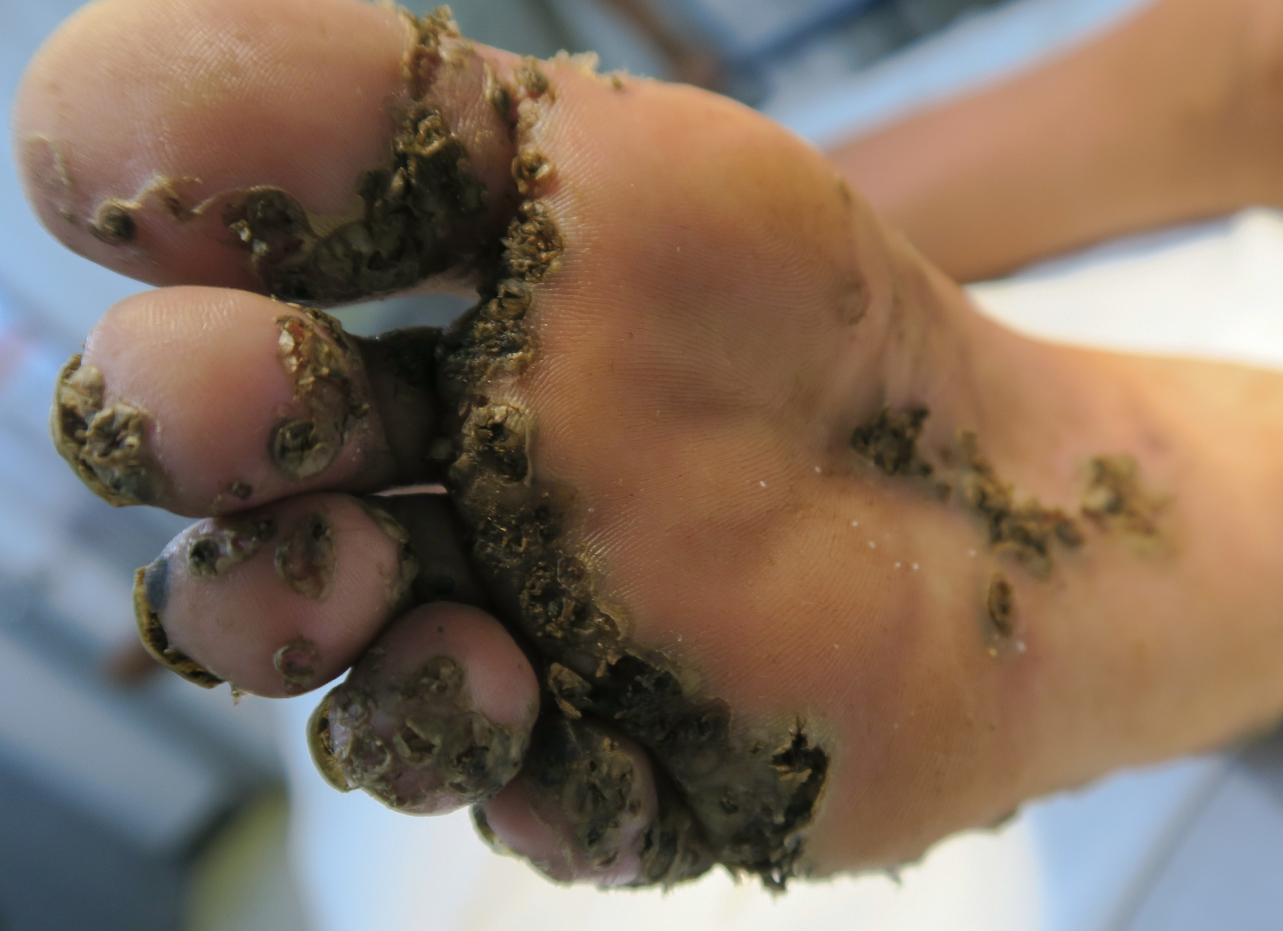
A cluster of embedded sand fleas at the sole below the toes and the interdigital area.

Patient 2 had approximately 250 embedded sand fleas in all stages of development of which the great majority had penetrated at the feet. The density of parasites was particularly high in the interdigital area of the soles (Fig 7). A small cluster of embedded sand fleas was detected around the anus. Most of the sand fleas were viable. The patient weighed only 23 kg.

Patient 4 had approximately 400 embedded sand fleas in all stages of development. Lesions were located on the soles, the lateral rim of the feet, ankles, knees, elbows and hands. On the soles lesions occurred in three layers on top of each other.

Patient 5 had approximately 1,000 lesions, of which 950 consisted of decaying or dead sand fleas at the time of examination.

### Laboratory findings

The laboratory findings are summarized in Table 2. Patient 1 and 3 had a severe anemia. Patient 2 had a leukocytosis of 12,700 cells/μl. Patient 2 and 4 showed a hypereosinophilia (2,540 and 2,520 eosinophils/μl, respectively).

**Table 2:** Hematological and other laboratory findings.

Parameter^a^	Patient 1	Patient 2	Patient 3	Patient 4	Patient 5
Erythrocytes (per μl)	3,310,000	5,060,000	2,360,000	3,120,000	3,500,000
Hemoglobin (g/dl)	7.8	13.9	5.8	10.0	8,1
Hematocrite (%)	24.5	44.2	17.3	29.2	26
MCV (fl)	74.2	87.5	37.7	93,8	74,5
MCH (pg)	23.5	27.4	24.5	32	23,1
Platelets (per μl)	146	445	265	342	248
Leukocytes (per μl)	4.9	12.7	5.4	7.2	5.7
Differential white blood cell count %					
neutrophils	72	44	70	41	60
lymphocytes	24	35	20	24	34
eosinophils	4	20	7	35	6
monocytes	0	0	0	0	0
basophils	0	1	2	0	0
Stool examination ^b^	*Not performed*	*hookworm spp*. *Entamoeba histolytica/ E*. *dispar/*	*hookworm spp*. *Chilomaxtis mesnilli*, *Trichomonas hominis*, *Entamoeba coli*, *Blastocystis hominis*,	*hookworm spp*. *Ascaris lumbricoides*, *Chilomaxtis mesnilli*, *Trichomonas hominis*, *Entamoeba coli*, *Blastocystis hominis*,	*hookworm spp*. *Chilomaxtis mesnilli*, *Trichomonas hominis*, *Entamoeba coli*, *Blastocystis hominis*,

^a^ Normal values: Erythrocytes 3,500,000–5,000,000 per μl; hemoglobin 11.0–15.0 g/dl; hematocrite 36.0–48.0%; MCV 80.0–99.0 fl; MCH 26.0–32.0 pg, platelets 150,000–450,000 per μl; leukocytes 5.0–10.0 per μl; differential white blood cell count: neutrophils 50–70%; lymphocytes 20–40%; eosinophils + basophils 1.0–15%

^b^ based on three microscopic examinations of a wet stool preparation

### Outcome

**Fig 8:**
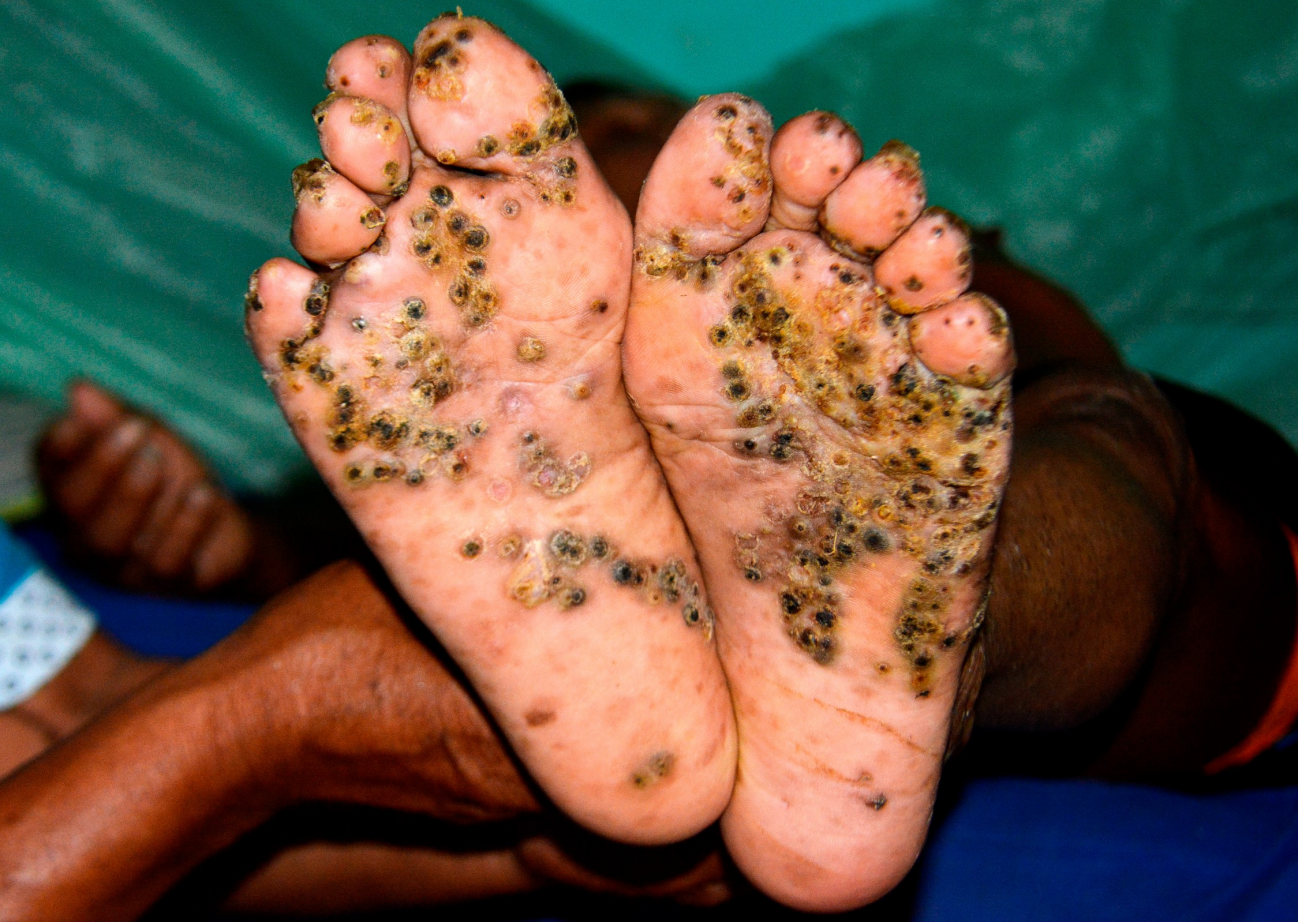
Soles one week after the first application of dimeticone. Parasites have died, the inflammation has decreased, and the skin has started to heal.

After two applications of the dimeticone oil, patients recovered rapidly. After three to four days, inflammation of the skin had regressed considerably (Fig 8) and patients could place their feet on the ground without feeling pain. After one week all lesions had developed into crusts and patients were transferred to a rehabilitation centre for Amerindians at the periphery of Mitú. At the end of the rehabilitation, i.e. 15 to 20 days after the first treatment with dimeticone, the patients had increased their weight Patient 1: from 35 to 41 kg; patient 2: from 23 to 28 kg; patient 3: from 39 to 45 kg; patient 4: from 33 to 41 kg (not data available from patient 5).

## Discussion

Literature from the middle of the 19^th^ to the middle of the 20^th^ century abounds with descriptions of very severe cases of tungiasis, in Latin America, the Caribbean as well as in sub-Saharan Africa [13–26, 30–34]. Since the 1950ties descriptions of very severe tungiasis became scanty. The few published cases concerned patients with alcohol addiction or psychiatric disorders who–at least from time to time–had been laying on the ground for hours [10, 12, 35, 36]. In current textbooks, very severe tungiasis is not mentioned at all.

This case series shows that very severe tungiasis still occurs, but goes unnoticed by health care providers because the patients are living in remote settings in the hinterland and do not have access to health care. The short period in which the five patients were identified indicates that very severe tungiasis is expected to occur in many Amerindian communities. Although this case series is small, it identified a pattern of characteristics which together determine that a self-limiting skin infection develops into a life threatening disease. Understanding of these determinants of very severe tungiasis allowed the development of a surveillance scheme by the Ministry of Health and Social Protection of Colombia.

### Factors predisposing to very severe tungiasis

It has been shown that age-specific prevalence curves show a peak in children and adults > 60 years and that the elderly always bear a high disease burden [1, 37]. Due to age-related poor sight elder people are unable to identify exactly where the parasite is located, and by consequence need help from family members to remove embedded sand fleas with a sharp instrument, the traditional treatment still in use in Amerindian communities [11]. Even if vision is adequate, elder people will usually be unable to bend down to the feet. Besides, elder people are less mobile and tend to stay at intradomiciliary transmission sites for many hours of the day. Another characteristic is that elder people frequently live alone and need to be cared for by relatives.

Patient 1 and 2 were wholly dependent on food provided by the only healthy adult person in the household. Patient 3, 4 and 5 were taken care for by their daughters. When the daughter left the community, the elder sons barely managed to provide food for their own family and usually nothing was left for the patients.

In Amerindians of the Amazon basin it is a question of survival for the family that old or handicapped people, who cannot provide food for themselves, become gradually separated from their families and are no longer cared for. This explains the malnutrition and cachexia observed in patients 1, 2 and 3.

Medical conditions which cause patients to involuntarily spend many hours in direct contact with the soil, such as sleeping sickness, mental disorders, alcoholism or Klippel-Trenaunay-Syndrome, are known since long as factors predisposing to very severe tungiasis [38, 39]. The same holds true when patients do not perceive pain or itch as in leprosy [38].

Patient 2 exposed skin of the buttocks for many hours of the day when crouching on his heels or squatting directly on a dirt floor in his ragged shorts. Persistently exposing skin at other areas than the feet facilitates the penetration of sand fleas at ectopic sites, such as around the anus and the inguinal area [13, 14, 22, 40].

Four patients had a pre-existing medical condition which restricted their mobility and left them laying in their hammock most time of the day. In the Amazon lowlands hammocks are installed such that they swing 20–40 cm above the floor. People rest in the hammock with a hand, an elbow, the lateral rim of the foot and/or the knee outside very close to the floor and place these body areas from time to time on the ground. By consequence, rather large areas of the skin are exposed to sand fleas which, in turn, explains the high parasite burden and the ectopic localizations (Figs 1B, 1C, 2B and 3B) [41, 42]. Since tungiasis of the feet impairs mobility due to severe pain, a vicious cycle develops in embedded sand fleas accumulating over time eventually resulting in very severe tungiasis and total immobility, if patients do not have access to treatment.

Another predisposing factor for a continuous accumulation of a high parasite load is intradomiciliary transmission. In the setting of the five patients, intradomiciliary transmission was very likely: In Amerindian communities, a fire is lit just below the hammock to warm up the sleeping person the whole night. This makes the surrounding earth floor dry and causes cracks in the soil, a perfect niche for the completion of the off-host cycle of *T*. *penetrans*. Dogs are frequently infected with *T*. *penetrans* and usually spend the night inside the dwelling next to the fire place [11, 13, 36]. Thus, eggs expelled from embedded sand fleas develop into adults in the area directly below the hammock or next to it. Hence, the probability to get infected is high as soon as the feet of a person are placed on the ground.

In Vaupés Amerindians strongly believe that severe tungiasis is due to an oath (*maldad* or *incantation*) and, hence, they are convinced that the disease cannot be cured. By consequence, people think that the afflicted person will be devoured by the parasite sooner or later and that only a traditional healer (*payé*) can interrupt this process. This explains the attitude that community members think that it is better to stay away from the affected person and why the patients of this study were secluded by the community.

**Fig 9:**
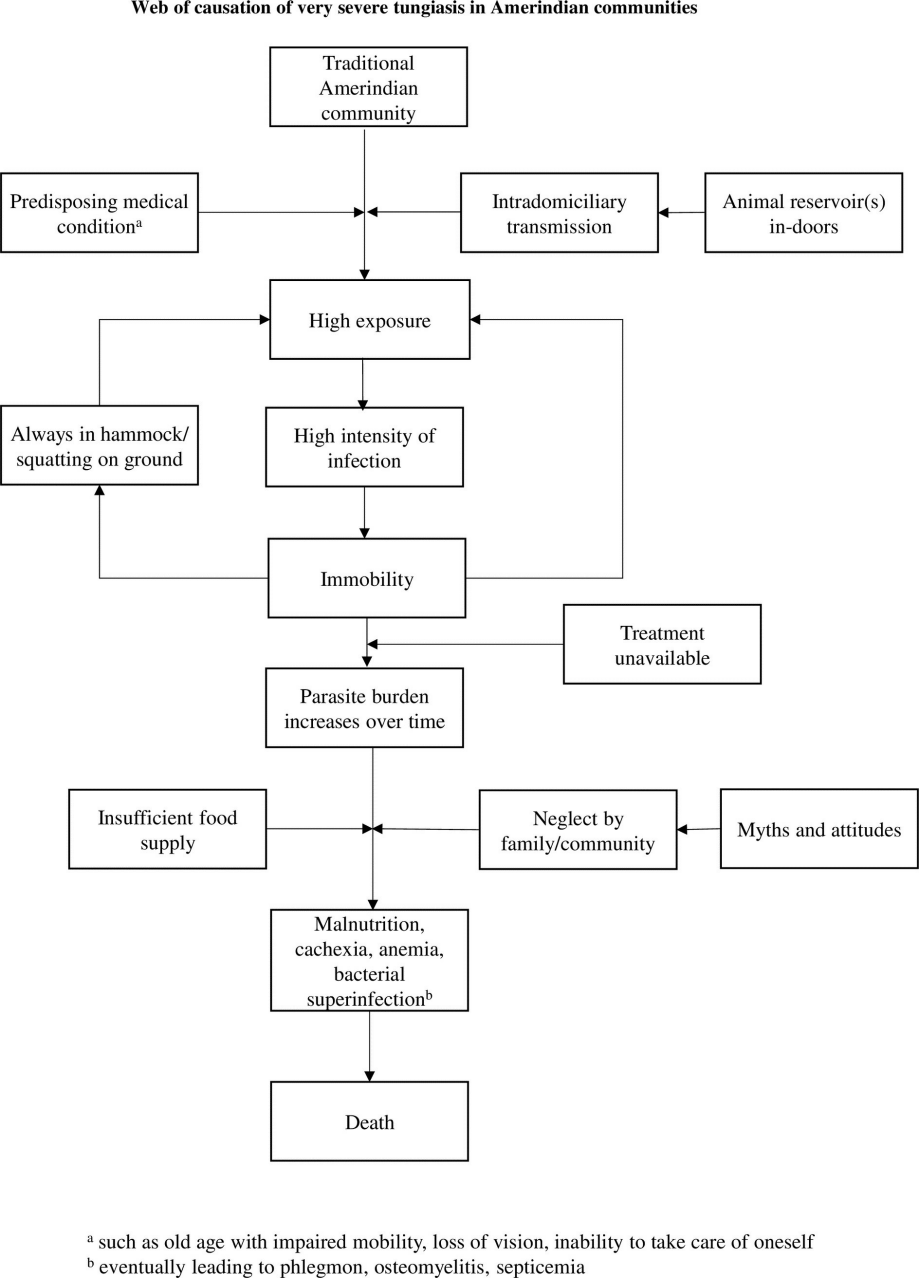
Web-of-causation making tungiasis a life-threatening condition.

The web-of-causation making tungiasis a life-threatening condition is depicted in Fig 9.

Lack of knowledge of the pathogenesis of tungiasis was identified as the leading cause of very severe disease and death in colonial times when Spanish and Portuguese conquistadors penetrated into the interior of the yet unknown continent and were confronted with pathogens they had never met before [43]. The scenario was similar in Africa, after *T*. *penetrans* arrived in Angola in 1875 and then rapidly spread along trading routes and with military missions [14, 17, 21, 23, 24, 33]; in natives of Madagascar after the disease all of a sudden appeared on the island [18, 25]; in troops deployed for the first time in an endemic area, such as French soldiers in Mexico in 1862 [16, 31].

That the lack of knowledge of tungiasis rapidly leads to very severe disease and eventually to death is best demonstrated by the history of 100 Irish settlers who moved from the Coast of French Guyana to the interior of the country in 1852. They became so heavily infected with sand fleas that 70 settlers died within a year and the remaining 30 came back to the coast in a very debilitated condition [25]. Lack of knowledge also seemed to play a role when tungiasis spread in Central Africa in the 1920s [33, 34]. However, lack of knowledge does not seem to play a role in the five patients. Tungiasis is known since pre-colonial times in Amerindians and local people clearly understand the pathogenesis of the disease [44].

### Morbidity

The spectrum of pathology associated with very severe tungiasis and its consequences are known since long. Already in 1900 L.L. Decle, a physician of the British army noted: “Ulcers caused by *Tunga penetrans* were the most frequent ailment treated and second only to smallpox as a cause of death in Luanda’s hospital between July and October 1877…” “Never in my life have I seen such awful ulcers. Some of the men had their bone of their big toe protruding fleshless for more than an inch; others had quite a square inch of the bone of the heel exposed. Even when tungiasis did not cause death, the parasite paralyzed movement.” The author continued:”In some villages of Uduhu, I found the people starving, as they were so rotten with ulcers from jiggers that they had been unable to work in their fields, and could not even go to cut the few bananas that had been growing.” [20]

Morbidity associated with tungiasis and its sequels is depicted in Table 3.

**Table 3:** Spectrum of morbidity and sequels in patients with very severe tungiasis.

Morbidity	Sequel
Deformation of feet (clubfoot-like)	Immobility -> malnutrition -> starvation -> cachexia
Mutilation of toes	Loss/amputation of toes
Deep fissure, ulcers with extended necrosis of tissue (*ulcère phagédénique*)	Immobility -> malnutrition -> starvation -> cachexia; bacterial superinfection
Abscess, suppuration, phlegmone	Lymphangitis, septicemia -> death
Infection with C*lostridia*	Tissue necrosis, gangrene, tetanus -> death
Hyperkeratosis of sole	Embedded sand fleas are located in several layers on top of each other and cannot be removed surgically; immobility due to extreme pain
Inflammation of feet	Oedema -> pain -> immobility
Osteomyelitis	Loss of toes and limbs -> death
Anemia	Incapacity to work and to collect food -> malnutrition -> cachexia; heart failure

The patients in this study showed a few peculiar findings. Patient 2 and 3 had a cluster of lesions around the anus, an ectopic localization which has never been described before.

Patient 1 and 3 showed a severe anemia which required immediate blood transfusion in patient 3. There is reason to believe that the anemia was the consequence of the high parasite load. First, severe infestation with *Ctenocephalides felis* in animals causes a significant anemia [45, 46]. Second, in contrast to most other *Siphonaptera*, which are only temporary ectoparasites, *T*. *penetrans* sucks blood almost permanently. Third, significant anemia was present in a very severe case of tungiasis from Tanzania with about 1,100 lesions [42]. With a persistent high parasite burden, the chronic blood loss will be substantial over time and eventually results in life-threatening anemia. Of course, the co-existing hookworm infection also may have contributed to anemia.

### Outcome

Hitherto, the only available treatment of tungiasis in Amerindian communities is surgical removal of embedded sand fleas using inappropriate instruments such as thorns, sharpened wooden sticks, knives etc. It goes without saying that this procedure is painful and always bears the risk of bacterial or fungal superinfection of the sore. Even in hospital settings, surgical extraction is virtually impossible if a great number of sand fleas are located in clusters or on top of each other in hyperkeratotic skin as in our patients.

The medical device NYDA contains two dimeticones with different viscosity and rapidly enters into tiny openings and covers microscopic surfaces [47]. The formula is used for the treatment of headlice in more than 20 countries. Its mode of action is purely physical. The topical application of this product has been proved highly effective in randomized control trials in Kenya and Uganda in patients with up to 30 embedded sand fleas [29, 48]. Here we show that the formula is also effective in patients with several hundred of embedded sand fleas located in clusters and in several layers on top of each other in hyperkeratotic or partially necrotizing skin. While in simple tungiasis an application of a few drops of the dimeticone—targeted to the abdominal cone which protrudes through the skin–are sufficient [48], in very severe tungiasis the skin needs to be intensively wetted and treatment should be repeated after 24 hours.

Taken together, this case-series shows that very severe tungiasis still occurs in Amerindian communities. The true frequency of this devastating condition is probably underestimated. A characteristic pattern of pre-existing medical conditions and socio-economic and environmental factors determines whether tungiasis develops into a life-threatening condition. Obviously, most of these factors are related to extreme poverty.

Our findings are also a good argument to make a call for action for those countries in which tungiasis occurs in remote settings and where health coverage is poor. Dimeticone should be made available to treat patients in an early stage of disease to avoid life-threatening sequels.
